# The Mediating Role of Procrastination in the Relationship between Fear of Missing Out and Internet Addiction in University Students

**DOI:** 10.3390/ijerph21010049

**Published:** 2023-12-29

**Authors:** Abdullah Manap, Amelia Rizzo, Abdullah Yıldırmaz, Ümit Dilekçi, Murat Yıldırım

**Affiliations:** 1 Department of Psychology, Batman University, Batman 72000, Türkiye; abdullah.manap@batman.edu.tr; 2 Department of Clinical and Experimental Medicine, University of Messina, 98100 Messina, Italy; amrizzo@unime.it; 3 Department of Cognitive Sciences, Psychological, Educational, and Cultural Studies, University of Messina, 98100 Messina, Italy; 4 Department of Public Relations, Batman University, Batman 72000, Türkiye; abdullah.yildirmaz@batman.edu.tr; 5 Department of Child Development, Batman University, Batman 72000, Türkiye; umit.dilekci@batman.edu.tr; 6 Department of Psychology, Faculty of Science and Letters, Agri Ibrahim Cecen University, Ağrı 04100, Türkiye; 7 Graduate Studies and Research, Lebanese American University, Beirut 1102 2801, Lebanon

**Keywords:** fear of missing out, internet addiction, procrastination, university students

## Abstract

This study aims to examine the potential mediating role of procrastination in the relationship between fear of missing out and internet addiction. Employing a cross-sectional research design, this study utilized a paper–pencil form to collect data from 315 students (65.4% undergraduate and 66.3% females) between 18 and 32 (Mage = 22.43; SD = 3.81) studying at a state university in Türkiye. Data were collected through the convenience sampling method. The study was designed as a survey model. This design was tested via mediation analysis. The participants completed self-report assessments using the Fear of Missing Out Scale (FoMOs), General Procrastination Scale (GPS-9), and Young Internet Addiction Scale (short form). The findings showed that FoMO had a significant positive direct effect on both procrastination (*R*^2^ = 0.13) and internet addiction (*R*^2^ = 0.33). Procrastination also had a significant positive direct effect on internet addiction (*R*^2^ = 0.34). Additionally, procrastination mediated the relationship between the fear of missing out and internet addiction (*β* = 0.156, *p* < 0.001). These findings not only extend the scope of existing research but also hold practical implications for the development of sustainable interventions. It is believed that the findings will contribute to the consideration of procrastination when preparing psychoeducation or group guidance programs for internet addiction. These interventions can effectively address the process through which fear of missing out leads to internet addiction by considering the significant role of procrastination in students. Findings typically suggest that procrastination behavior is a key factor in explaining the association between FoMO and internet addiction.

## 1. Introduction

The increasing use of the internet around the world has brought about a number of positive impacts, including increased access to information and educational opportunities and the development of global communication [1]. As of June 2021, the internet penetration rate across the world had climbed to 67.9%, which is approximately 1392% higher than the rate during the last two decades [2]. This increasing rate causes certain problems, such as online security problems and privacy concerns, and especially internet addiction, to emerge more frequently [3]. In addition, the widespread use of electronic devices that provide access to the internet has aroused interest in studies on the impacts of internet use on individuals [4,5]. Due to the widespread use of the internet, constant interaction, notably through social media platforms, has become an important factor that can deepen individuals’ fear of missing out (FoMO). 

FoMO is defined as a pervasive concern that others may have rewarding experiences in the absence of oneself [6]. Students’ FoMO has led them to use the internet more intensively [7]. Students experiencing FoMO may exhibit behaviors that are likely to pose a risk of addiction due to constantly checking social media with the desire to see what others are doing and to have the same experiences as them [8]. It has been stated that the internet and social media have a large role in FoMO becoming a widespread concern among individuals [9]. Therefore, the internet is considered to be a good distraction and a facilitator of procrastination [10]. In this respect, it can be said that FoMO plays a vital role as a factor that is likely to trigger internet addiction among students along with the increase in the use of the internet. 

Internet addiction is characterized by behaviors associated with the use of the internet in an uncontrolled way [4]. As a result, it may lead to problems in individuals’ functionality of their daily routines. According to the related literature, in certain studies on internet addiction, the excessive use of the internet has been associated with procrastination behaviors, which is related to pedagogical problems [11,12,13,14]. Procrastination behavior is viewed as the tendency to postpone or avoid decisions and tasks to the extent that they cause negative consequences [11]. According to Gong et al., procrastination affects internet addiction [15]. This may lead individuals with a tendency to procrastinate to exhibit behaviors that may increase the risk of addiction due to the use of the internet as an escape mechanism. FoMO, on the other hand, is likely to prevent individuals from using their time efficiently, causing them to postpone their tasks [16]. In this regard, it has been acknowledged that there is a complex relationship between FoMO, internet addiction, and procrastination behavior. To this end, the research provides a framework for understanding the relationship between FoMO, internet addiction, and procrastination behavior. 

### 1.1. Fear of Missing Out

Fear of missing out (FoMO) is a term used to denote the view that when individuals grapple with the feeling that they are missing out, they experience a common concern that other people may be having fun in their absence and a desire for obligation for following what other people are doing [6,17]. FoMO was first conceptualized in the literature as a field of psychopathology since it is associated with the anxiety caused by perceived anxiety related to psychosocial need deficits, such as the need to belong and socialize [8,18]. Although the term was first conceptualized in an offline or real-world context, it has also been dealt with in terms of social media use [6]. Researchers in this field also emphasize that the impact of FoMO on the lives of individuals needs to be further investigated [8,19]. 

FoMO may exert significant pressure on the internal state of the individual. This pressure can influence individuals’ lives by encouraging them to stay continually connected with the information that is constantly being shared through social media [8,20]. In this respect, it has been revealed that the increase in the use of social media triggers the anxiety of users to socialize and to fall behind the new experiences and opportunities [6,21]. The authors defined this term in their previous studies as ‘others might be having rewarding experiences from which one is absent’. In addition, with the increased interest in social media, it has been observed that FoMO has gained more power and, therefore, the feeling that individuals’ own lives are incomplete has been mediated more easily than ever before [9]. An experimental analysis of the effect of FoMO on individuals found that of all participants who sat with or without a mobile phone in a room alone, the ones without mobile phones experienced a higher level of anxiety during a seven-minute waiting period [22]. Numerous experimental studies in this field similarly prove that FoMO may lead to the overuse of social media [6].

### 1.2. Internet Addiction

Healthy internet use refers to the use of the internet for a specific purpose at an appropriate time, without any cognitive or behavioral impairment [23]. However, problematic internet use can be defined as the individuals’ inability to control the continuous use of the internet due to problems such as family and work experiences [24]. It can be said that the number of problematic internet users is high nowadays [25]. In the related literature, researchers have described this as ‘Internet addiction’ [26,27], ‘pathological Internet use’ [28,29], and ‘problematic Internet use’ [30,31,32].

Internet addiction is defined as the loss of control over the use of the internet by individuals, and it is stated that this addiction leads to various problems and social incompatibilities in the daily lives of individuals [4,33]. Even though the ease of access to information is seen as an advantage of internet use, researchers have often found that excessive internet usage causes internet addiction, yielding negative outcomes in terms of individuals’ personal, financial, and professional lives [4]. There is a growing body of literature studying the addictive use of the internet and the factors resulting in internet addiction [34,35,36,37,38,39].

The fact that the internet allows a person to easily communicate with other people without assuming any responsibility by hiding one’s identity poses a significant risk for addiction [40,41]. Moreover, through the digital transformations that have occurred globally in recent years, individuals use the internet more frequently [42]. As a result, the internet has become a medium that puts many people at risk of developing a behavioral addiction. Additionally, it has been stated that the risk of developing a behavioral addiction is higher during adolescence compared to other age groups [43,44]. Students are one of the main groups most subjected to this addictive impact of the internet [13]. Many studies show that in most countries, students spend more than two hours a day on the internet [45,46,47]. This intensive use of the internet also has several negative outcomes [13]. Compared to the students who use the internet in a controlled way, the ones using the internet intensively have certain difficulties in their academic performance and daily routines [48]. Nevertheless, the effects of these difficulties may vary depending on how and for what purposes students use the internet, as well as how much control they have in terms of the amount of time they use the internet [49,50]. 

### 1.3. Procrastination Behaviour

Individuals occasionally tend to procrastinate in their daily lives, and this happens in a way that the individual focuses on other things instead of a task that needs to be performed immediately or leaves the task for a while [10]. Postponed tasks are often perceived by individuals as less urgent or less enjoyable, and individuals are likely to gravitate towards activities that provide instant gratification or that seem more appealing [51].

There is an accumulated body of literature on the approach in which procrastination behavior and personality traits are evaluated together, suggesting that there is a correlation between procrastination behavior and personality traits of the individual [52]. This approach asserts that personality traits such as extraversion, low conscientiousness, and external locus of control are associated with a tendency to procrastinate [53]. Situational procrastination is a behavior that occurs under the influence of internal or external factors. According to this approach, individuals may be prone to procrastinating as those tasks offer less fun or unpleasant experiences for them [54]. Said differently, provided that a task becomes too difficult or boring, it will be perceived as unpleasant by individuals and avoided as a result. In the same vein, when an individual experiences anxiety as a result of an assessment, s/he feels a lack of control over completing a task, and procrastination behavior may occur [55]. Furthermore, it has been alleged that procrastination is indirectly related to time management issues [10]. It has been observed that those who procrastinate often know exactly what they need to do, yet they postpone this task. The frequent and uncontrolled continuation of procrastination behavior may also adversely affect the functionality of the person in their daily routines [56]. Accumulating evidence provides support for the fact that procrastination is linked to lower performance and negative business outcomes [53]. These behaviors might increase the stress levels of individuals who have the habit of procrastinating and negatively influence their productivity [57].

### 1.4. The Effect of FoMO on Internet Addiction

People with high levels of FoMO constantly ask to stay connected with others and keep track of what others are doing [6]. As a platform that allows users to be in constant communication with their peer groups, social media may offer an outlet for individuals to reduce their FoMO levels [8]. 

Many studies have been conducted to explore the relationship between FoMO and internet addiction [6,19,58]. Studies examining the relationship between the two concepts often address the effect of the use of the internet and social media on FoMO. In [59], the authors carried out a study revealing that internet use increased FoMO and posited that the internet was likely to trigger FoMO in individuals by constantly providing the opportunity to see the activities and experiences of others through social media platforms and other online communication tools. On the contrary, it should be noted that the relationship between FoMO and internet use is bidirectional. In other words, while internet use increases FoMO, FoMO can also lead individuals to greater levels of internet use [19]. This situation can be witnessed in a way that individuals are consistently checking social media and participating in online interactions due to the fear of missing out on the activities of others [60]. Findings from various research fields have indicated that FoMO is associated with internet addiction, including smartphones, social media, and games. In [61,62], the authors concluded that there was a significant relationship between high levels of FoMO and internet addiction. 

### 1.5. The Mediating Role of Procrastination

In the literature, there is a growing number of studies suggesting that there is a robust relationship between FoMO and procrastination [16,63,64]. Individuals with FoMO feel the need to constantly check and participate in social media for FoMO on other people’s social activities [6]. This can trigger procrastination behavior by preventing them from diverting their time and attention to other things [65]. In this context, FoMO can lead individuals to postpone tasks that need to be fulfilled [63]. Further research on this topic is thought to provide a deeper insight into the relationship between FoMO and procrastination behavior.

Considering the prevalence of the internet nowadays, internet-based tools are also the most accessible tools for procrastination [51]. Individuals can carry out activities such as maintaining social relationships, communicating with friends, receiving new information, or accessing entertaining content through the internet. These activities may lead individuals to postpone their tasks by offering instant gratification [66]. There are also studies in the literature that argue that there is no significant relationship between procrastination and internet addiction [67,68]. However, several current studies confirm the robust relationship between internet use and procrastination [42,44,69]. In a study by Lavoie and Pychyl [51] examining the associations between internet addiction and procrastination, it was reported that more than half of the participants frequently procrastinated due to the internet. Likewise, Davis et al. noted that procrastination was conceptualized as a key aspect of problematic internet use [30]. Viewed from this perspective, it is stated that there is a positive relationship between procrastination and problematic internet use [13]. Although comprehensive studies have been conducted to investigate this topic in Europe and the United States, little has been said about these concepts in Türkiye. In light of the preceding discussion, the present study is devoted to exploring the linear relationship between FoMO and internet addiction by considering procrastination as the mediating variable.

### 1.6. Present Study

This study seeks to address an important research gap in the existing literature by investigating the relationship between FoMO, procrastination, and internet addiction among Turkish young adults. While prior research has explored individual aspects of these phenomena, there remains a distinct lack of understanding regarding how FoMO may contribute to internet addiction and the potential mediating role of procrastination in this relationship. This study aims to fill this knowledge deficiency by employing a regression-based mediation analysis approach [70] to test the mediating role of procrastination in the relationship between FoMO and internet addiction. The findings are anticipated to present scientific evidence about the underlying mechanism between FoMO, procrastination, and internet addiction, providing a foundation for more targeted interventions and strategies to address these issues among the young adult population. The hypotheses tested within the scope of the research were as follows:

**H_1_.**  *FoMO would have a significant positive effect on both procrastination and internet addiction. That is, individuals with FoMO are expected to exhibit procrastination behavior, and at the same time, these individuals are expected to view the internet as an escape area and exhibit behaviors that may pose a risk of addiction*.

**H_2_.**  *Procrastination would have a significant positive effect on internet addiction. In other words, it is expected that individuals with procrastination behavior tend to engage more in using the internet*.

**H_3_.**  *Procrastination would mediate the relationship between FoMO on internet addiction. That is to say, individuals with high levels of FoMO tend to have procrastination behaviors, which in turn has the potential to lead to internet addiction behaviors*.

## 2. Materials and Methods

### 2.1. Participants

Using a cross-sectional research design, the present study was conducted on 315 students studying at Batman University in Türkiye. The participants consist of 66.3% females and 33.7% males. Among them, 34.6% are studying at the associate degree level, while 65.4% are at the undergraduate level. The age range of the participants varies between 18 and 32, with a mean age of 22.43 (SD = 3.81).

### 2.2. Measures

The Fear of Missing Out Scale (FoMOs) was developed by Zhang et al. [71] and validated in Turkish by Çelik and Özkara [72]. The scale aims to measure individuals’ levels of FoMO on the developments they perceive on social media. The elevated scores on the scale suggest high levels of FoMO among the participants, while the lower scores indicate a corresponding lower level of FoMO. The scale score ranges from a minimum score of 9 to a maximum of 63. The FoMOs includes 9 items clustered into two subscales: Personal FoMO (5 items) and Social FoMO (4 items). Each item is rated using a 7-point Likert-type scale, ranging from 1 (strongly disagree) to 7 (strongly agree). A sample item is “I feel anxious when I’m not active on social media”. Çelik and Özkara’s [72] validity and reliability analyses revealed a Cronbach alpha coefficient of 0.90 for Personal FoMO and 0.89 for Social FoMO. Additionally, the model’s fit index values were determined as follows: χ^2^/df = 2.58, RMSEA = 0.07, SRMR = 0.08, and CFI = 0.90. In this study, Cronbach’s alpha coefficient was found to be 0.93. 

The General Procrastination Scale (GPS-9) is the short form of Lay’s General Procrastination Scale [73] and was developed by Sirois et al. [74]. A validity and reliability study of the Turkish version was conducted by Şimşir Gökalp et al. [75]. The GPS-9 includes 9 items, and each item is answered based on a 5-point Likert-type scale ranging from “strongly agree” to “strongly disagree”. A sample item is “I generally delay before starting work I have to do”. The scale aims to measure individuals’ level of procrastinating responsibilities. Elevated scores on the scale highlight heightened levels of procrastination behaviors among participants, while lower scores indicate a corresponding lower propensity for procrastination. The scale score ranges from a minimum score of 9 to a maximum of 45. In the validity and reliability analyses conducted by Şimşir Gökalp et al. [75], the Cronbach alpha coefficient was established at 0.87. The model’s fit index values are as follows: χ^2^/df = 4.232, GFI = 0.94, CFI = 0.94, and RMSEA = 0.09. In this study, Cronbach’s alpha coefficient was found to be 0.85.

The Young Internet Addiction Scale (short form) was developed by Kimberly Young [76] and adapted into a short form by Pawlikowski et al. [77]. A validity and reliability analysis of the Turkish version was conducted by Kutlu et al. [78]. The scale consists of 12 items rated on a 5-point Likert-type scale ranging from “rarely” to “always”. A sample item is “How often do you lose sleep due to being online late at night?” The scale aims to measure the perceived levels of internet addiction of individuals. High scores on the scale suggest heightened levels of internet addiction among participants, while lower scores indicate a corresponding lower susceptibility to internet addiction. Kutlu et al.’s [78] validity and reliability analyses show a Cronbach alpha coefficient of 0.85. The model’s fit index values are present as follows: χ^2^/df = 3.275, CFI = 0.95, RMSEA = 0.07. In this study, Cronbach’s alpha coefficient was found to be 0.89.

### 2.3. Procedure

Initially, requisite permissions were secured from the researchers through email to employ the scales for this study. Then, approval was received from the Batman University Ethics Committee (E-131160/2023-05-20). Data were gathered using the pencil-and-paper method, and the package of questionnaires contained information text, personal information questions, and scales. Informed consent was obtained from participants before collecting data, and they were assured of confidentiality and anonymity of responses. Participants voluntarily took part in the study without receiving any incentives. The scales were delivered to university students from different departments to maximize sample diversity (18 May–23 June/2023). Data were collected online using a convenience sampling method.

### 2.4. Data Analysis

The normality assumption of the data was examined by analyzing skewness and kurtosis scores [79]. Based on the skewness and kurtosis normality tests, data distribution was found to be within an acceptable range. Pearson correlation analysis was performed to explore relationships between variables. Then, the proposed mediation model (Model 4) was tested using the PROCESS v4.0 for Windows. Mediation analyses aim to measure the mediating effect of a third variable on the effect of the independent variable on the dependent variable. The results from Model 4 provide information about whether the mediator significantly influences the relationship between the independent variable and the dependent variable. It helps researchers understand whether a third factor (mediator) could explain the relationship between the independent variable and the dependent variable [70]. In this study, a mediating variable (procrastination) was examined in terms of the effect of the independent variable (FoMO) on the dependent variable (internet addiction). In the related body of literature, no research was encountered that empirically investigates these relationships. Therefore, this model (Model 4) was chosen to make inferences about the relationship between FoMO, internet addiction, and procrastination behavior. All statistical analyses were performed by using SPSS version 26. 

## 3. Results

According to the findings, the data are normally distributed based on skewness and kurtosis values [79]. Skewness ranges from 0.267 to 1.668, and kurtosis ranges from −0.165 to 3.451. According to Kim [80], these values are considered to represent a normal distribution. The normal distribution of the data indicates that there is no problem with extreme values, and further analysis can be conducted. 

The correlation findings between FoMO, procrastination, and internet addiction are presented in Table 1. FoMO showed a positive correlation with procrastination (*r* = 0.365, *p* < 0.001) and with internet addiction (*r* = 0.573, *p* < 0.001). Procrastination was positively correlated with internet addiction (*r* = 0.580, *p* < 0.001). According to the correlation analysis, there are moderate-level significant and positive relationships between FoMO, procrastination, and internet addiction. The correlation analysis results show significant relationships between variables, referring that regression analysis can be performed.

The results of the analysis regarding the mediation role of procrastination in the effect of FoMO on internet addiction are given in Table 2. According to the findings, FoMO directly and significantly affected procrastination (*β* = 0.250, *p* < 0.001) and internet addiction (*β* = 0.262, *p* < 0.001). FoMO explained 13% of the variance in procrastination and 33% of the variance in internet addiction. Additionally, procrastination directly and significantly affected internet addiction (*β* = 0.394, *p* < 0.001). Procrastination explained 34% of the variance in procrastination. Accordingly, all of the direct effects were positive and significant.

**Figure 1:**
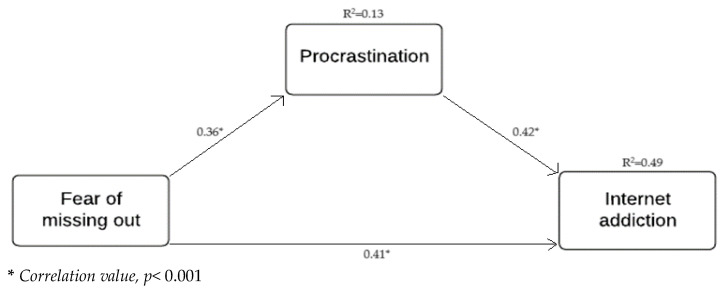
Proposed model indicating the standardized coefficients between variables.

When the mediation effect of procrastination on the effect of FoMO on internet addiction was examined, it was seen that the level of indirect effect was positive and significant. It was concluded that BootLLCI and BootULCI values did not contain 0. FoMO and procrastination together explain 49% of the variance in internet addiction. As a result, it can be said that procrastination had a mediating role in the effect of FoMO on internet addiction (*β* = 0.156, *p* < 0.001). Results obtained from the study show that higher levels of FoMO and procrastination are indicative of higher levels of internet addiction. Furthermore, procrastination mediated the relationship between the fear of missing out and internet addiction (see Table 2 and Figure 1). As a result, procrastination behavior partially mediated the relationship between FoMO and internet addiction, and it has also been determined that the high level of FoMO and procrastination behavior is related to the high level of internet addiction.

## 4. Discussion

Based on the findings obtained within the scope of this research, which aims to examine the mediating role of procrastination in the relationship between FoMO and internet addiction, there is a significant correlation between FoMO, procrastination behavior, and internet addiction. A positive and significant relationship between FoMO and procrastination behavior and internet addiction has been detected. Additionally, there is a positive and significant relationship between procrastination behavior and internet addiction. It has been shown that as the level of one variable of all variables, which are FoMO, procrastination, and internet addiction, increases, the level of other variables also increases. According to the body of related literature, it has been found that procrastination behavior affects internet addiction. In a research conducted by Gong et al., it is stated that procrastination plays a mediating role between perceived stress and internet addiction [15].

The first hypothesis of the research is that *“Fear of missing out in university students would have a significant positive effect on both procrastination and internet addiction”.* In light of the findings, it has been indicated that the FoMO levels of university students have a direct, positive, and significant effect on their procrastination behavior and internet addiction. In other words, as the FoMO levels of university students increase, their tendency to exhibit procrastination behavior also increases. Additionally, as the FoMO levels of university students increase, their internet addiction levels also increase. In the related literature, no study directly investigating the relationship between FoMO and procrastination behavior has been encountered. Some correlations and interactions can be detected between these two variables, yet further research is needed to explain this relationship more precisely. Contrary to this, there are studies designed to explore the relationship between FoMO and internet addiction in the literature. The fear of individuals who follow social media to deal with the negative emotions caused by FoMO and to feel better [81] also increases the use of social media [6]. Accordingly, the non-functional and uncontrolled use of problematic internet may be triggered by the tendency of individuals not to miss potentially better alternatives for social interaction [82]. This can lead to higher levels of problematic social network usage symptoms [83]. The finding that FoMO significantly predicts internet addiction concurs in certain studies, suggesting that the increase in the use of social media tools may result from FoMO [6,7,17,84]. According to the findings of research intended to explore the relationship between internet addiction, FoMO, and psychological factors, it has been observed that individuals who experience higher levels of FoMO are more likely to participate in social media [85]. Nowadays, with the prevalence of social media, students may feel that their own lives are inadequate or incomplete as they see the content shared on social media platforms, thereby leading them to constantly check social media platforms. Persistent exposure to content shared on social media platforms can have negative effects on students. Most importantly, it can make it difficult for the students to focus on their training. Therefore, it can be stated that it is vital for students to balance between their education and the internet and to use social media consciously.

The second hypothesis of the research is that “*Procrastination would have a significant positive effect on Internet addiction*”. The findings indicated that the procrastination behavior of university students has a direct, positive, and significant effect on their internet addiction. To put it differently, as the procrastination behaviors of university students increase, their internet addiction levels also increase. This finding concurs with the findings of prior studies in the related literature [30], noting that there is a correlation between procrastination and problematic internet use. In this regard, excessive use of the internet may be attributed to some psychological or emotional factors. These behaviors often emerge as an escape mechanism [51]. Similarly, it is emphasized that students who often procrastinate show a tendency to problematic internet use to a greater extent [86]. Students can use the internet in an attempt to avoid or commence challenging tasks, posing a risk of addiction [67]. This situation leads to internet addiction over time for students who use the internet intensively. This addiction, however, may negatively affect students’ academic achievements, social relationships, and physical health, causing them to lose their balance in their daily lives. As a result, it is of great importance for students to manage the duration of their use of the internet in a balanced way and to adopt healthier strategies to deal with challenging tasks.

The third hypothesis of the research is that “*Procrastination would mediate the relationship between fear of missing out on Internet addiction*”. The research findings have demonstrated that procrastination has a mediating role in the effect of FoMO levels of university students on their internet addiction. In other words, it has been observed that the FoMO level of university students is associated with their procrastination behaviors and plays a mediating role in the effect of these procrastination behaviors on internet addiction. It is also reported in the introduction of the study that FoMO has a positive effect on internet addiction. Studies examining the relationship between these variables argue that people’s psychological desire to follow others and FoMO leads them to use social media more frequently [6,19,58]. On the other hand, this poses the risk of developing addiction for individuals [87]. Consequently, FoMO, which is considered a psychological syndrome related to excessive use of the internet, can result in internet addiction [88]. The mediating effect of procrastination on the effect of FoMO on internet addiction can be supported by studies on procrastination behavior. In a study carried out by Wang et al. [63] investigating the relationship between the level of smartphone addiction and thrill-seeking behavior, it was concluded that procrastination influenced the relationship between thrill-seeking and smartphone addiction as a mediating factor. Based on the findings of another study, it was found that procrastination had a mediating role between problematic smartphone use and adolescent depression [89]. In light of the abovementioned studies, it can be said that procrastination has behavioral effects on individuals. Given the fact that FoMO, internet addiction, and procrastination have a mutual effect on one another, it also seems theoretically likely that procrastination might play a mediating role in FoMO’s impact on internet addiction. In this respect, students may tend to spend more time on social media by postponing important tasks when they experience FoMO [90,91]. Therefore, it is thought that procrastination may increase the effect of students’ FoMO levels on their internet addiction by having a mediating role. Students experiencing FoMO may postpone their real-life tasks since they want to participate in online activities and stay up-to-date on the internet. Therefore, FoMO has the potential to cause procrastination behavior by influencing students’ habits of internet use. This is thought to increase the risk of internet addiction in the long run.

## 5. Implication and Limitations

The internet provides an interactive environment for students and poses the risk of developing addictive behaviors by pulling them toward this environment. Based on the findings of this study, FoMO may lead students to tend towards online environments offered by the internet, causing them to exhibit an addiction-level use and postpone the tasks that they should initially perform. In addition, since students can use the internet for different purposes, identifying which purpose may be more problematic than others will be interesting for further studies. In recent years, further research has been needed to better understand the antecedents of increasing internet addiction and to develop strategies to combat addiction. In addition, research can be carried out to identify the use of the internet for various purposes such as education, entertainment, and interaction with other individuals and also to detect possible problems. The findings obtained from the study can be useful for educational scientists, psychologists, psychiatrists, and therapists both in preventing behaviors that may cause addiction and in developing online addiction therapies. This has important implications for young individuals [92]. 

The current study has certain limitations. The findings of the study with cross-sectional design do not reveal causation. The data collection on independent, mediator, and dependent variables from a single source in the research can be shown as another limitation of the research. This may cause the problem of common method variance. In the study, the data were obtained from only one university. Consequently, research data are limited in terms of student profiles and diversity. Considering that the ability of the sample to represent the universe is limited, it is thought that researchers can contribute to the literature by collecting data from different populations for further studies. Considering that the current study is a cross-sectional study, a direct causal relationship between the variables can not be revealed. Internet addiction may have several causes other than FoMO and procrastination behaviors. This research is limited by the effect of these two variables on internet addiction. On the other hand, procrastination was tested as a mediator between FoMO and internet addiction when designing the research. It is also thought that further studies will be useful to test other important variables concerning the relationship between FoMO and internet addiction that may predict this relationship and affect students’ academic achievement. 

Along with the existing data, the findings yielded by the research are also considered important in terms of the measures to be taken to reduce the internet addiction of students. For this reason, to develop effective and powerful strategies while combating internet addiction, it is important to understand procrastination behavior and to create educational programs that raise awareness of this issue. Such educational programs are thought to play an effective role in combating internet addiction by helping students improve their time management skills and reduce their tendency to procrastinate. To understand students, face-to-face interviews can be conducted individually, or the needs of the students can be determined through psychometric questionnaires. Campaigns, seminars, and workshops to raise awareness can be organized in schools aligned with counseling and guidance services. By carrying out group therapies or support groups, students can be encouraged to share their experiences. Activities can be arranged for teachers, school administrators, and other influential people to be good role models in the use of the internet. In order to ensure that the activities listed above are sustainable, long-term goals can be set, and fundraising strategies can be created in cooperation with institutions. In this regard, it is expected that the findings of the research will contribute to the consideration of procrastination behavior in the design of psychoeducation and group guidance programs in schools. While this research identifies a positive relationship between FoMO, procrastination behavior, and internet addiction, it is also highly important to consider the purposes for which students use the internet since the effects of online activities carried out by students for different purposes on internet addiction may also be different. Further research is needed to distinguish between students’ purposes of internet use. This is important to understand the possible risk factors and protective factors depending on the students’ intended internet use. In this context, it is thought that the research will shed light on future longitudinal studies. For future research, the mediating role of different variables in the relationship between FoMO and internet addiction can be examined. Internet addiction is a complicated issue that requires researchers from different disciplines, such as education, psychology, and sociology, to come together and study it. For further research, it is recommended to adopt a multi-disciplinary approach combining the perspectives of different disciplines such as education, psychology, and sociology. 

## 6. Conclusions

This study aims to examine the potential mediating role of procrastination in the relationship between FoMO and internet addiction. This is because there is no research in the literature that tests these relationships empirically. The findings showed that FoMO had a significant positive direct effect on both procrastination and internet addiction. Procrastination also had a significant positive direct effect on internet addiction. Additionally, procrastination mediated the relationship between the fear of missing out and internet addiction. This study is expected to contribute to other studies on FoMO, procrastination behaviour, and internet addiction.

## Figures and Tables

**Table 1:** Descriptive statistics and correlation analysis.

	Description Statistics					Correlation Coefficients (*r*)		
Variable	α	Min.	Max.	M	SD	1.	2.	3.
1. Fear of missing out	0.93	1	7	2.05	1.12			
2. Procrastination	0.85	1	5	2.57	0.77	0.37 *		
3. Internet addiction	0.89	1	5	2.14	0.71	0.57 *	0.58 *	

* *p* < 0.001.

**Table 2:** Testing the pathways of the mediation model.

Path						
Unstandardized Direct Effect	R^2^	F	*β*	*SE*	LLCI	ULCI
Fear of Missing Out > Procrastination	0.13	48.14	0.250 *	0.03	0.179	0.321
Fear of Missing Out > Internet Addiction	0.33	152.8	0.263 *	0.02	0.208	0.317
Procrastination > Internet Addiction	0.34	158.8	0.394 *	0.04	0.315	0.473
Completely Standardized Indirect Effect(s)					BootLLCI	BootULCI
Fear of Missing Out > Procrastination > Internet Addiction	0.49	148.1	0.156 *	0.024	0.1089	0.2047

* *p* < 0.001, LLCI = The lower limit confidence interval ULCI = The upper limit confidence interval FoMO = Fear of Missing Out, Pro = Procrastination, IA = Internet Addiction.

## Data Availability

The data supporting this study’s findings are available from the corresponding author upon reasonable request.
